# Mapping the Neural Dynamics of Korean–English Bilinguals With Medium Proficiency During Auditory Word Processing

**DOI:** 10.3389/fpsyg.2018.00983

**Published:** 2018-06-18

**Authors:** JeYoung Jung, Kichun Nam, Hyesuk Cho, Sunmi Kim

**Affiliations:** ^1^Neuroscience and Aphasia Research Unit, Division of Neuroscience and Experimental Psychology, School of Biological Sciences, The University of Manchester, Manchester, United Kingdom; ^2^Department of Psychology, Korea University, Seoul, South Korea; ^3^Wisdom Science Center, Korea University, Seoul, South Korea

**Keywords:** bilinguals, functional connectivity, neural plasticity, dorsal attention network, default mode network (DMN), fMRI

## Abstract

Bilingualism is a worldwide phenomenon and provides an opportunity to understand how the brain represents language processing. Although many studies have investigated the neural mechanism of bilingualism, it still remain unclear how brain systems are involved in the second language processing. Here, we examined the neural dynamics of bilinguals with medium proficiency during auditory word processing. Korean–English (K–E) bilinguals were recruited for the study (L1: Korean and L2: English). They performed a word comprehension task on phonological and semantic aspects by hearing words. We compared their task performance, task-induced regional activity, and functional connectivity (FC) between L1 and L2 processing. Brain activation analyses revealed that L2 evoked more widespread and stronger activation in brain regions involved in auditory word processing and the increased regional activity in L2 was prominent during phonological processing. Moreover, L2 evoked up-regulation during semantic processing was associated with L2 proficiency. FC analyses demonstrated that the intra-network connectivity showed stronger in the language network (LN), dorsal attention network (DAN), and default mode network (DMN) in L2 than L1. For the L2 phonological processing, the increased FC within the DAN was positively correlated with individuals’ L2 proficiency. Also, L2 semantic processing induced the enhanced internetwork connectivity between the LN and DMN. Our findings suggest that L2 processing in K–E bilinguals induces dynamic changes in the brain at a regional and network-level and FC analysis can disentangle the different networks involvement in L2 auditory word processing according to two key features: phonology and semantics.

## Introduction

Bilingualism is a worldwide phenomenon and more than half of the world’s population is estimated as bilinguals (Grosjean, 2010). Moreover, modern education systems often provide a second language teaching from childhood since world become more connected and societies have realized the necessity of multilingualism to communicate with each other globally. In step with this global phenomenon, there have been many studies about learning more than one language linguistically and psychologically. However, little is known about how bilingualism influences brain function yet. Only recently, it has become one of key topics in neuroscience to unveil the neural mechanism of bilingualism.

Functional neuroimaging studies have investigated the neural correlates of languages in bilinguals (for the review, see Abutalebi et al., 2001; Kroll and Groot, 2005; Stowe, 2005; Indefrey, 2006; Abutalebi, 2008; Sebastian et al., 2011). These reviews suggest several general findings. First, L2 and L1 processing seems to be processed in a language network (LN) including the left inferior frontal gyrus (IFG), insular, anterior cingulate cortex (ACC), dorsolateral prefrontal cortex (DLPFC), superior and middle temporal gyri (STG/MTG), the angular gyrus, and temporal pole (Kim et al., 1997; Perani et al., 2003; Wartenburger et al., 2003; Jones et al., 2012). Specifically, bilinguals with high proficiency show similar activation in the same language system for L2 and L1 processing, whereas bilinguals with medium/low proficiency exhibit a more extended network of activation, broadening activation into the right hemisphere for L2 processing. Second, a common network contributes to the semantic processing for L1 and L2 (Chee et al., 1999; Ruschemeyer et al., 2005), whereas somewhat different neural networks is involved in syntactic processing for L1 and L2, especially for bilinguals with late L2 onset (Wartenburger et al., 2003; Ruschemeyer et al., 2005). For the syntactic processing, neural differences between L1 and L2 are prominent in the early stage of L2 acquisition and/or bilinguals with low proficiency. When bilinguals become more proficient in L2, these differences between L1 and L2 disappear, suggesting the neural plasticity reflected the change in language processing. These findings indicate that the brain plasticity of bilingualism can be modulated with the age of L2 acquisition, the level of L2 proficiency, and the amount of L2 experience. Here, we investigated the neural mechanism of Korean–English bilinguals during auditory word compression by controlling these factors.

In addition to the language system, there are other several brain networks involved in L2 processing. The examination of bilingualism has focused on executive control network (ECN) (Bialystok et al., 2012). The network is involved in several interrelated processes such as inhibition, updating, and shifting, consisting of the dorsolateral/inferior frontal cortex and inferior parietal cortex (Duncan, 2010; Spreng et al., 2013). Thus, this domain-general system is required in bilinguals to switch between languages or suppress their L1 while speaking in L2 or vice versa (Abutalebi et al., 2001; Abutalebi, 2008). Recent fMRI studies have showed that not only the ECN but also other domain general networks are associated with L2 processing (For the review, see Pliatsikas and Luk, 2016). These include the salience network (SN), including the anterior insular, the dorsal anterior cingulate cortex (dACC), and the supramarginal gyrus (SMG) (Seeley et al., 2007), the dorsal attention network (DAN) including dorsolateral prefrontal cortex (DLPFC), frontal eye fields, inferior precentral sulcus, and superior parietal lobule (Corbetta and Shulman, 2002), and the default mode network (DMN), consisting of the posterior cingulate cortex (PCC), the ventromedial prefrontal cortex, the angular gyri and the parahippocampal gyri (Fox et al., 2017). Grady et al. (2015) compared resting state connectivity between older bilinguals and monolinguals by focusing on three resting state networks (ECN, SN, and DMN). They found the stronger functional connectivity (FC) in bilinguals for the ECN and DMN, but not for the SN compared to monolinguals. Another study (Li et al., 2015) compared Chinese bimodal bilinguals to monolinguals. They examined brain regions related to both sign and spoken language processing and domain general regions (dACC and left caudate). They reported the reduced FC between the dACC and superior temporal gyrus (STG) in bimodal bilinguals than monolinguals. Cao et al. (2014) recruited the late Chinese–English bilinguals to investigate the FC in L1 and L2 rhyming judgment. They found that Chinese rhyming judgment increased the FC between visual-orthographic regions and the right precentral gyrus, whereas English processing was associated with the greater FC between visual-orthographic regions and the left postcentral gyrus. Importantly, this enhanced FC in English processing was positively correlated with their English proficiency. These studies suggest that L2 processing in bilinguals modulates the intra-and internetwork connectivity in various brain networks including task-specific and domain general systems. However, it is not well understood how multiple brain networks and their integration support the L2 processing in bilinguals.

Korean–English (K–E) bilinguals are highly homogeneous bilinguals sharing ethnicity and culture and having similar educational backgrounds for L2 learning. English has always been the first foreign language in South Korea and English curriculum has started since 1950s. Korean people spent $15.6 billion on English education including all extracurricular lessons such as private tutoring, English camp, and short-term language training abroad (Chun and Choi, 2006). This phenomenon was called ‘English fever’ and now English in South Korea become ‘a class marker’ (Park and Abelmann, 2002). However, considering the incredible social demand, only few studies examined the neural representations in K–E bilinguals (Kim et al., 1997; Lee et al., 2002; Jeong et al., 2007a,b; Suh et al., 2007; Cho et al., 2009). Jeong et al. (2007a,b) studied sentence comprehension in Korean and Chinese speakers with their L1 and two L2 (English and Japanese). During English sentence comprehension compared to L1, Korean bilinguals showed greater activation in the left IFG and bilateral STG than Chinese bilinguals. During Japanese sentence processing compared to L1, the Chinese group showed greater activation in the left anterior STG than the Korean group. Another study (Suh et al., 2007) examined the syntactic processing of sentence comprehension in Korean–English bilinguals. They found that the left IFG, IPL and occipital cortex were activated for both L1 and L2 but only the left IFG showed the interaction effect between language and sentence type (embedded vs. conjoined). Cho et al. (2009) investigated brain activation in Korean-English and Korean–French high proficiency bilinguals during lexical judgment and picture naming. They reported the increased activation in the left IFG and right prefrontal cortex during L2 processing compared to L1, regardless of L2 languages. Consistent with other bilinguals’ findings, these studies reported that the same language system was shared for L1 and L2, with more activation for L2 processing and Korean specific effects were reported in the left language related regions during sentence level processing. Studies with K–E bilinguals have focused on the brain representation and the level of regional activity for L1 and L2. However, a growing body of studies suggests that bilingualism is associated with large-scale differences in brain networks and it is important to understand how dynamic coherence of neural network is influenced by L2 processing. Here, we investigate similarities and differences in brain activation and FC in L1 and L2 auditory comprehension in K–E bilinguals. Especially, we accessed both phonological and semantic processing to tackle key aspects of auditory word comprehension and to provide convergent understanding of the neural mechanism in K–E bilinguals.

In the current study, we investigate the neural dynamics of K–E bilinguals with medium L2 proficiency using fMRI. Bilinguals performed a phonological task and semantic task by hearing words in L1 and L2. We compared their task performance, task-induced regional activity, and task-induced FC between L1 and L2 processing. As medium-proficiency bilinguals, they would perform the both tasks better in L1 than L2. Also, we expected that the auditory word processing regions would be activated for both L1 and L2, but the level of activation would be greater for L2. Previous studies demonstrated strong correlations between increased activity in cortical regions and the language processing (Price, 2000; Friederici, 2002). In order to explore the relationship between the level of cortical activity and L2 ability, we performed correlation analysis between the regional activity and individuals’ English score. Finally, to evaluate the L2 processing at brain network-level, we conducted FC analysis in five brain networks including the LN and other domain general networks (ECN, SN, DAN, and DMN). We expected that FC analysis would reveal differences between L1 and L2 processing, which cannot be detected in brain regional analysis. We also examined how L2 ability was associated with FC during L2 processing. We expected that high English score would be related with the increased FC during L2 processing.

## Materials and Methods

### Participants

Sixteen healthy, right-handed volunteers (7 males, averaged age: 23.4 ± 2.5 years; mean laterality quotient: 80.8) participated in this study. All participants were native Korean speakers and acquired English as the second language from the public education at the age 9.9 ± 1.6. All participants were asked to assess their English skills with 5 points Likert scale (1 – very poor, 2 – poor, 3 – average, 4 – good, 5 – very good) and to provide official English scores such as Test of English for International Communication (TOEIC), Test of English as a Foreign Language (TOFLE) and Test of English Proficiency developed by Seoul National University (TEPS). They gave their written consent in accordance with national legislation and the Helsinki Declaration. This study was approved by the Ethical Committee of the Korea University.

### Stimulus and Experimental Design

One hundred and ninety four high frequency nouns were selected based on phonological and semantic features each for L1 and L2 from a Korean pronunciation and an English dictionary. We conducted a familiarity questionnaire with 7 points Likert scale for the selected words. Thirty Korean native speakers answered the questionnaire. They also evaluated the emotional valence of each word with three choices (negative, neutral and positive). A total of 60 nouns scored highpoint (Korean: 6.79 ± 1.17, English: 6.59 ± 1.85) at the questionnaire were chosen for each language and recorded by a male and a female native speakers (L1: Korean, L2: English) (see Supplementary Table S1). All nouns consisted of two syllables. The half of nouns had a long vowel (L1) or a lexical stress (L2) at the first syllable. Korean has a vowel-length contrast in words (Kim-Renaud, 1994). Such long vowels can typically occur in the first syllable of a word. In this study, we selected two syllable nouns having a long vowel in their first syllable. The half of nouns for the semantic judgment task had positive meaning and the rest negative meaning. The average duration of words was 1.08 s ± 0.23 s for L1 and 1.12 s ± 0.39 s for L2 and there was no difference between them.

**FIGURE 1:**
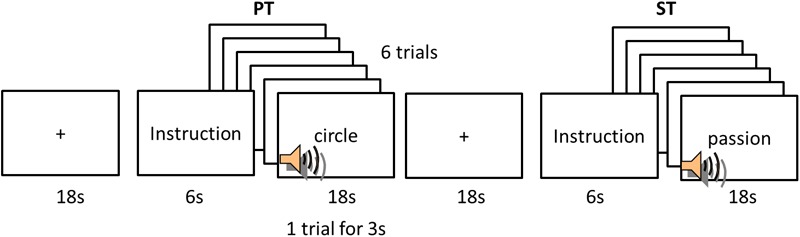
Experimental design. PT, phonological task; ST, semantic task.

To tackle two key aspects of word comprehension, we employed long vowel judgment/lexical stress judgments and emotional valence judgment for L1 and L2. For the purpose of the current study, we refer to them as phonological and semantic judgment tasks. But it is noted that these judgments are involved in not only phonological and semantic processing but also other aspects of language processing (e.g., phonetic, acoustic, emotion, etc.). Participants performed two tasks for each language: phonological judgment task (PT) and semantic judgment task (ST). A block-design fMRI was used and a session consisted of 5 blocks of PT and 5 blocks of ST interspersed with 10 fixations (Fix) for each language (**Figure 1**). Each block was preceded by a fixation (8 s) before the 6 s instruction. The instruction indicated which task participants should perform in a following block. Following the instruction, a word was auditorily presented without any presentation on the screen. There were 6 stimuli in each task block. A word was presented in 3 s and the duration of a block was 18 s. During the fixation, participants were asked to fixate on a cross at the center of the screen. In the PT, participants listened to a word and pressed the first button with the index finger of their right hand if a heard word has a lexical stress (L2) or a long vowel (L1) in the first syllable and the second button with the right middle finger if it did not. In the ST, participants listened to a word and decided the emotional valence of words (positive vs. negative). If the meaning was positive, they had to press the first button with the right index finger and the second button for negative meaning. The order of blocks was counterbalanced. E-Prime software was used to present the experiment and to record responses.

### fMRI Data Acquisition and Analysis

3T Siemens scanner was used to acquire imaging data at the Korea University Magnetic Resonance Imaging Centre. Anatomical images were acquired using MPRAGE sequence (TR = 1900 ms, TE = 2.52 ms, flip angle = 9°, matrix = 256 × 256, resolution = 1 mm × 1 mm × 1 mm) covering the whole head. Functional images were obtained using single-shot echo planer imaging (EPI) sequence (TR = 2000, TE = 30, flip angle = 90°, number of slices = 36, matrix = 80 × 80, 3 mm × 3 mm × 4 mm).

SPM8 package (Wellcome Department of Cognitive Neurology, United Kingdom) was used to analyze the fMRI data. Functional images were realigned (motion correction), co-registered with individual anatomical images, spatially normalized to the Montreal Neurological Institute (MNI) space, and spatially smoothed using a Gaussian kernel (8 mm, Full-width half-maximal). Statistical analyses were performed using a general linear model (GLM). Four contrasts for each language were computed for each participant (PT > Fix, ST > Fix, PT > ST, ST > PT). In random-effects analyses, contrast images of each subject were used for one-sample *t*-tests to identify regions activated by tasks and paired *t*-tests to examine language specific regions according to the tasks. Statistical threshold was set at *p* < 0.001 uncorrected at the voxel-level and the resulting images were assessed for cluster-wise significance (*p* < 0.05 FDR-corrected for multiple comparisons) with a spatial extent threshold of at least 50 contiguous voxels.

Region of Interest (ROI) analysis was employed to assess the level of activation in key language regions. Based on the GLM results (the contrast of PT > Fix and ST > Fix), five ROIs were selected bilaterally: inferior frontal gyrus (IFG), superior temporal gyrus (STG), supramarginal gyrus (SMG), inferior parietal lobe/inferior parietal sulcus (IPL/IPS), and supplementary motor cortex (SMA). To define ROIs, we used the templates from WFU_PickAtlas Toolbox^1^ for each ROI (**Figure 4**).

### Functional Connectivity Analysis

We employed the Functional Connectivity (CONN) Toolbox^2^ to perform the FC analysis. To examine the intra- and inter-connectivity changes between L1 and L2, five well-known functional networks were selected: LN, ECN, SN, DAN, and DMN. The LN consists of IFG and posterior parietal-temporal cortex; the EN includes DLPFC and posterior parietal cortex (PPC); the DAN comprises of the FEF and IPS; and the DMN includes the medial PFC (mPFC), precuenus/PCC, and angular gyrus (AG). The networks were defined from CONN’s independent component analyses (ICA) of Human Connectome Project (HCP) dataset. For the inter-network connectivity, we examined the FC between the LN and the other domain general networks.

Pre-processed images were entered to the toolbox. Data were filtered using a band pass filter (0.01 < f < 2) to decrease the effect of low-frequency drift. White matter, cerebrospinal fluid, and physiological noise source reduction were taken as confounds, following the implemented CompCor strategy (Behzadi et al., 2007). Head motion was taken into account and rotational and translational motion parameters and their first-order temporal derivatives were regressed out. The onset and duration of each experimental condition was supplied to the toolbox so as to extract the connectivity generated for L1 and L2 during phonological and semantic processing. In the first-level analysis, a voxel-to-voxel correlation map was produced for each subject per each condition. This was done by extracting the corresponding residual blood oxygenation level-dependent (BOLD) time course from a voxel and computing Pearson’s correlation coefficients between that time course and the time course of all other voxels. To examine the FC in the networks, ROI-to-ROI analysis was performed by grouping voxels into ROIs in a network. The BOLD signal time course was averaged from all voxels compromising each ROI. Bivariate correlations were calculated between each pair of ROIs as reflections of connections. ROI-to-ROI analyses were performed for all subjects’ data with a GLM to extract task-specific connections at the individual level. The intra-network connectivity was calculated by averaging correlation coefficients between the ROIs within the network and the inter-network connectivity were estimated by averaging them between the networks. The estimated FCs were analyzed by 2 × 2 ANOVA with task (PT vs. ST) and language (L1 vs. L2). To link the FCs and individual’s L2 score, correlation analysis was performed.

## Results

### Characteristics of Bilinguals and Behavioral Results

All participants have learned English as L2 at age 10, for about 15 years, ranging from 7 to 13. The average education they had was about 15 years ranging from 13 to 8 years. They assessed their English skills as an average level (score around 3, see **Table 1**). Their official English score was 838.7 ± 107.4 ranging from 625 to 975 (the other test scores were translated into TOEIC score). The TOEIC consists of Listening and Reading section and the perfect score is 990 (Listening: 495; Reading: 495). According to TOEIC score guidance, their English level was ‘working proficiency plus level,’ which satisfies most work requirements with language that is often, but not always, acceptable and effective.

**Table 1:** Characteristics of bilinguals.

Age of acquisition	Education (years)	English self-assessments			English score (TOEIC)
		Listening	Speaking	Writing	
9.9 (1.7)	14.8 (1.2)	3.4 (0.7)	2.8 (1.0)	3.2 (0.8)	838.7 (107.4)

*All values represent mean (Standard deviation).*

**FIGURE 2:**
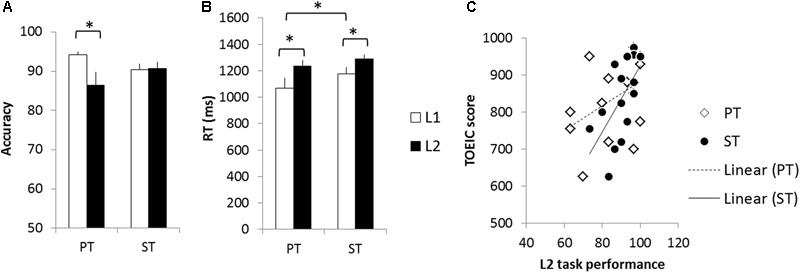
Behavioral results. **(A)** Accuracy. **(B)** Reaction time. **(C)** Correlations between task performance and TOEIC score. White bars represent the L1 performance and black bars the L2 performance. Error bar indicates the standard error. White diamonds indicate the L2 PT accuracy and black circle L2 ST accuracy. ^∗^*p* < 0.05.

Participants performed the PT and ST for both languages. Only trials in which participants correctly responded were included in the analyses. A repeated-measure ANOVA with language (L1 vs. L2) and task (PT vs. ST) as within-subjects factors was conducted in accuracy and reaction time (RT). In the accuracy, there was a significant interaction between language and task (*F*_1,15_ = 10.77, *p* < 0.005) and the other effects did not reach the significance (*p*s > 0.16). *Post hoc* paired *t*-tests revealed that participants made more errors in L2 PT than L1 (*t* = 2.42, *p* < 0.05) (**Figure 2A**). RT analyses revealed a significant main effect of language (*F*_1,15_ = 12.43, *p* < 0.05) and task (*F*_1,15_ = 5.15, *p* < 0.05). There was no significant interaction (*p* = 0.30). *Post hoc* tests demonstrated that participants were significantly slower in L2 for the both tasks than L1 (PT: *t* = 3.23, *p* < 0.05; ST *t* = 2.60, *p* < 0.05) (**Figure 2B**). Also, participants performed the PT faster than the ST in L1 (*t* = 3.24, *p* < 0.05). In order to examine the relationship between their L2 task performance and TOEIC score, correlation analyses were performed. The L2 accuracy was positively correlated with the TOEIC score (PT: *r* = 0.42, *p* = 0.058; ST: *r* = 0.61, *p* < 0.01) (**Figure 2C**).

### fMRI Results

**FIGURE 3:**
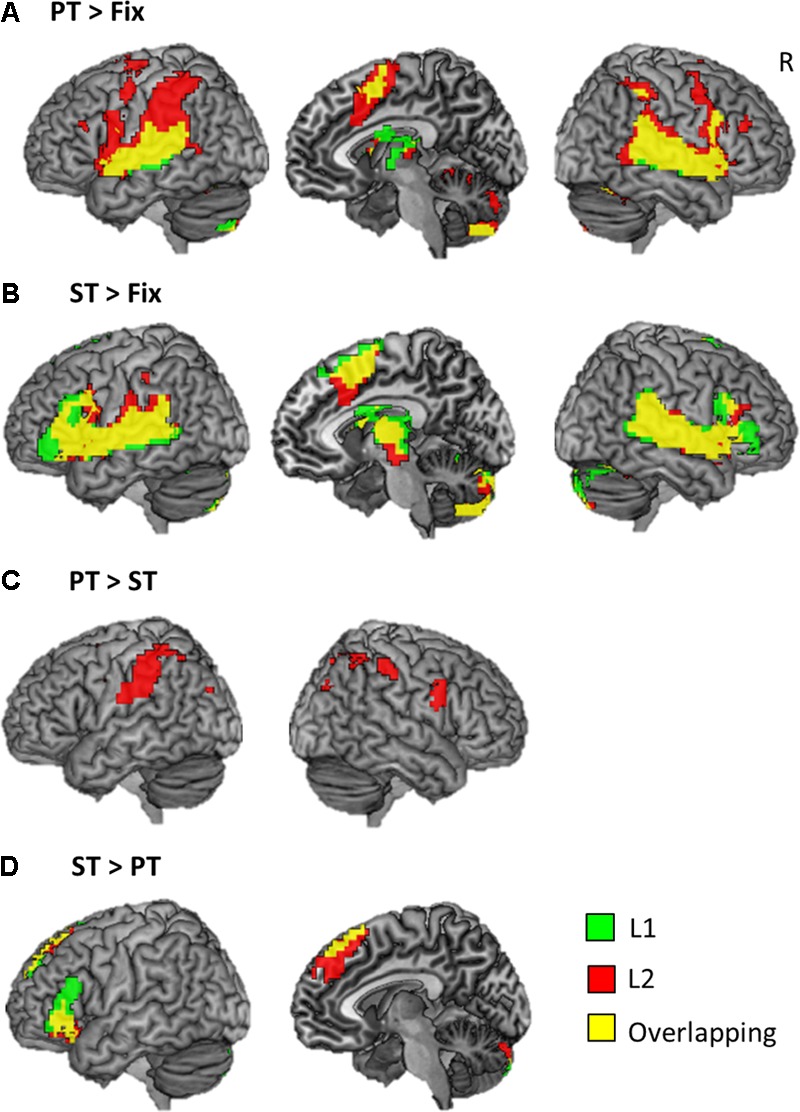
Brain activation maps. **(A)** The results of phonological processing. **(B)** The results of semantic processing. **(C)** The comparison of PT > ST. **(D)** The comparison of ST > PT. Green color represents the result of L1 processing, red the results of L2 processing, and yellow overlapping.

The whole brain analysis revealed that each task evoked significant activation in the frontal, temporal, and parietal cortex bilaterally across the languages (**Figure 3** and **Table 2**). Both PT and ST increased activation in the IFG, STG/MTG, IPL, and SMA (**Figures 3A,B**). L2 processing seems to recruit more widespread activation in the same regions. In the contrast of PT > ST, we found a significant activation in the bilateral SMG, IPL/IPS, right precentral gyrus and right SFG only for L2 (**Figure 3C**), whereas, in the ST > PT contrast, there was a significant activation in the left IFG and superior frontal gyrus (SFG) for both languages (**Figure 3D**).

**Table 2:** Results of whole brain analyses.

Language	Region	Laterality	*x*	*Y*	*z*	*t*
L1	**PT > Fix**					
	STG	R	60	-19	2	10.52
		L	-54	-13	2	9.49
		L	-48	-4	-6	6.58
	MTG	L	-66	-31	6	6.24
	SMA	L	0	8	54	5.28
		L	-6	-1	70	4.34
		L	-12	2	58	4.14
	**ST > Fix**					
	STG	R	54	-22	2	12.98
		L	-54	-16	2	10.5
	Putamen	L	-24	-1	14	9.78
	SMA	L	-3	8	54	6.86
		L	-3	23	58	4.04
	mSFG	L	0	35	54	5.15
	**PT > ST**					
	-					
	**ST > PT**					
	IFG	L	-51	32	-2	6.12
		L	-57	29	14	5.28
		L	-48	14	14	5.27
	mSFG	L	-6	41	54	5.8
	SFG	L	-12	56	30	4.97
	SMA	L	-3	23	62	4.05
	MTG	L	-42	-43	6	5.54
		L	-63	-40	6	4.13
		L	-42	-31	2	3.65
L2	**PT > Fix**					
	STG	R	57	-19	2	13.62
		L	-54	-10	2	10.96
	Insular	L	-33	17	2	12.82
	SMA	R	9	8	50	9.64
		L	-3	5	54	9.51
	Thalamus	L	-15	-19	6	10.96
	**ST > Fix**					
	STG	R	51	-16	2	12.2
		L	-54	-37	10	13.28
	SMA	R	9	14	46	8.61
		R	12	5	66	5.28
	SFG	L	-12	17	42	6.58
	IPL	L	-39	-43	42	3.65
		L	-30	-43	42	3.43
		L	-33	-34	38	3.24
	**PT > ST**					
	SMG	L	-54	-34	34	11.5
		R	45	-34	38	7.82
	IPL	L	-57	-31	46	8.08
		R	54	-31	50	7.08
	AG	R	30	-58	42	6.3
	Precentral gyrus	R	60	8	34	6.46
	SFG	R	30	2	62	5.08
	Insular	R	39	-7	14	4.81
	MFG	L	-24	2	58	6.15
		L	-27	-10	50	4.44
	SMA	L	-12	2	66	4.87
	**ST > PT**					
	IFG	L	-54	29	2	8.09
		L	-33	26	-10	7.94
		L	-39	35	-10	6.99
	mSFG	L	0	41	46	6.98
		L	-9	41	34	6.9
	SFG	L	-12	29	58	6.32
	MTG	L	-63	-40	2	4.75
	ITG	L	-51	-19	-22	4.71

*STG, superior temporal gyrus; MTG, middle temporal gyrus; SMA, supplementary motor area; IFG, inferior frontal gyrus; SFG, superior frontal gyrus; mSFG, medial SFG; IPL, inferior parietal lobule; SMG, supramarginal gyrus; AG, angular gyrus; MFG, middle frontal gyrus; ITG, inferior temporal gyrus.*

**FIGURE 4:**
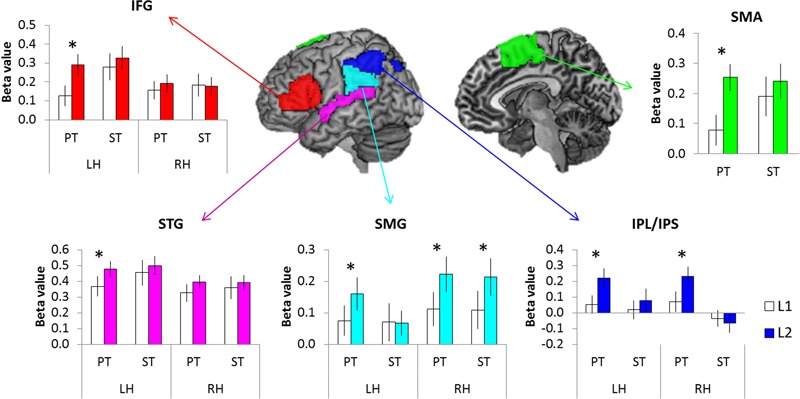
The results of ROI analysis. White bars represent the L1 processing and color bars the L2 processing. Error bar indicates the standard error. ^∗^*p* < 0.05.

We conducted the ROI analysis to examine the effect of language and task in the brain regions activated for auditory word processing. The regional activity in the IFG, STG, SMG, IPL/IPS, and SMA were estimated according to experimental conditions. A repeated-measure ANOVA with language (L1 vs. L2), task (PT vs. ST), and hemisphere (LH vs. RH) was conducted for each ROI. The results revealed that L2 tasks increased regional activity in these regions compared to L1, especially during the PT (**Figure 4**). The IFG showed a significant main effect of language (*F*_1,15_ = 6.34, *p* < 0.05) and interaction between task and hemisphere (*F*_1,15_ = 8.44, *p* < 0.05). The regional activity in the STS showed a significant main effect of hemisphere (*F*_1,15_ = 5.63, *p* < 0.05) and interaction between language and hemisphere (*F*_1,15_ = 4.92, *p* < 0.05). The SMG revealed a significant main effect of language (*F*_1,15_ = 5.99, *p* < 0.05) and interaction between language and hemisphere (*F*_1,15_ = 13.21, *p* < 0.01). The IPL/IPS also showed a main effect of language (*F*_1,15_ = 3.56, *p* = 0.05) and interaction between language and hemisphere (*F*_1,15_ = 15.67, *p* < 0.001) as well as between task and hemisphere (*F*_1,15_ = 6.50, *p* < 0.05). The SMA showed a significant language effect (*F*_1,15_ = 10.40, *p* < 0.01). *Post hoc* tests demonstrated that L2 PT induced the significant up-regulation in the left IFG, left STG, bilateral SMG, bilateral IPL/IPS, and SMA. The right SMG showed the increased activation during L2 ST.

**FIGURE 5:**
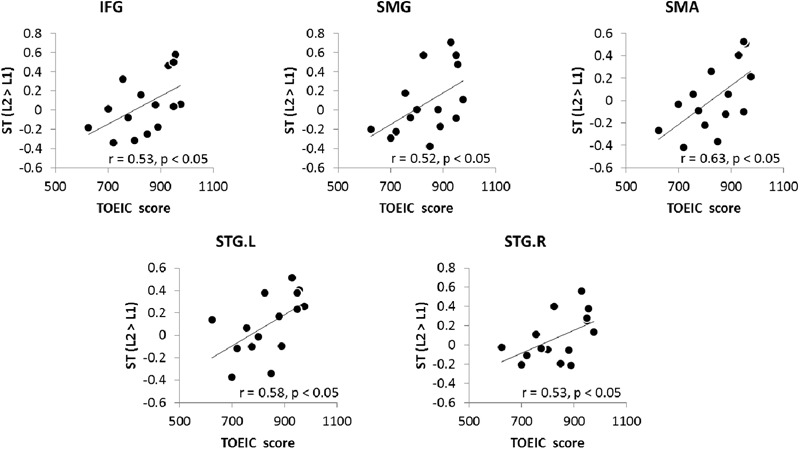
The relationship between the TOEIC score and increased activation during L2 ST.

In order to investigate whether the up-regulation found in the ROIs contributes to bilinguals’ L2 proficiency, correlation analyses was conducted between the TOEIC score and the regional activity in the contrast of L2 > L1. There were significant correlations between the TOEIC scores and ROIs’ activity during ST in the left IFG, SMG, SMA and bilateral STG (p _FDR-corrected_ < 0.05, one-tailed) (**Figure 5**). Stronger activity in these regions was associated with better L2 proficiency in bilinguals with medium proficiency.

### FC Results

In order to examine the intra- and inter-network connectivity between L1 and L2, we performed the FC analysis in the five brain networks (LN, ECN, SN, DAN, and DMN). The Pearson’ correlation coefficients were estimated between the key regions within a network for the intra-network connectivity and the regions between networks for the inter-network connectivity. Then, they were averaged individually, according to the languages and tasks.

**FIGURE 6:**
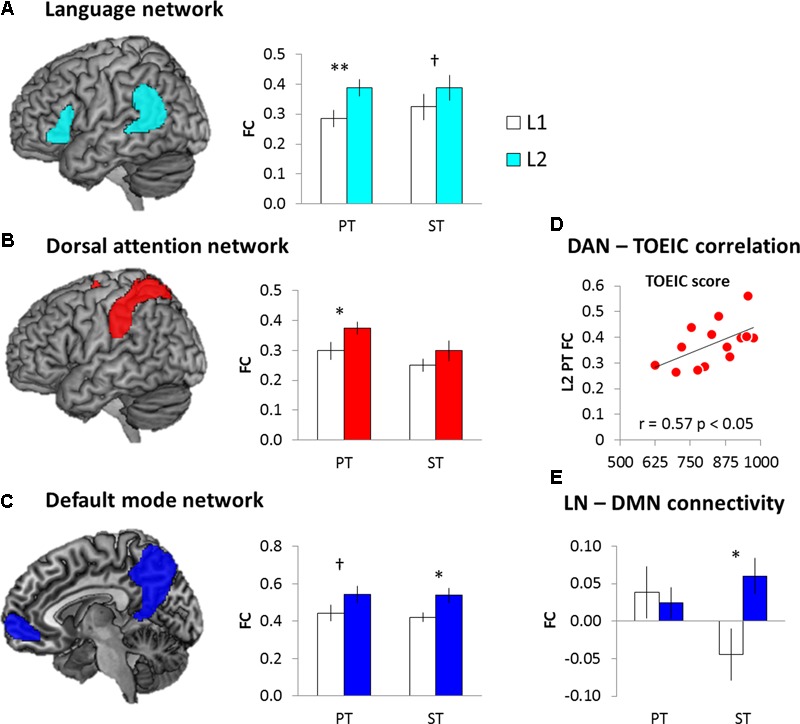
The results of FC analysis. **(A)** Language network. **(B)** Dorsal attention network. **(C)** Default mode network. **(D)** DAN-TOEIC correlation during the L2 PT. **(E)** Internetwork connectivity between the LN and DMN. White bars represent the L1 processing and color bars the L2 processing. Error bar indicates the standard error. ^∗^*p* < 0.05, ^∗∗^*p* < 0.01, ^†^*p* = 0.1.

For the intra-network connectivity, 2 × 2 ANOVA with task (PT vs. ST) and language (L1 vs. L2) was conducted for each network. The results demonstrated that there was a significant main effect of language in the LN (*F*_1,15_ = 8.97, *p* < 0.01), DAN (*F*_1,15_ = 5.96, *p* < 0.05), and DMN (*F*_1,15_ = 7.55, *p* < 0.05). *Post hoc* paired *t*-tests showed that L2 induced significantly increased intra-connectivity in the LN (PT: *t* = 3.78, *p* < 0.005; ST: *t* = 1.30, *p* = 0.10), DAN (PT: *t* = 2.15, *p* < 0.05; ST: *t* = 0.12, *p* = 0.80), and DMN (PT: *t* = 1.90, *p* = 0.07; ST: *t* = 2.12, *p* = 0.05) (**Figures 6A–C**). The other networks did not show significant FC changes. Then, we investigated the relationship between the L2 proficiency and intra-network connectivity during L2 processing. We found that the increased FC in the DAN during the L2 PT was significantly correlated with the TOEIC score (**Figure 6D**). Bilinguals with stronger FC in the DAN achieved higher score in the official English test.

In order to investigate the internetwork connectivity, we examined the FC between the LN and the other networks. 2 × 2 ANOVA with task (PT vs. ST) and language (L1 vs. L2) was conducted. The results showed that there was a significant interaction between the language and task only in the LN-DMN connectivity (*F*_1,15_ = 4.81, *p* < 0.05). *Post hoc* paired *t*-tests showed that there was the increased internetwork connectivity between the LN and DMN during the L2 semantic processing (**Figure 6E**).

## Discussion

The present study examined different measures of dynamic activity in the brains of K–E bilinguals. We evaluated both phonological and semantic processing in L1 and L2 during auditory word comprehension. Behaviorally, K–E bilinguals with medium proficiency performed poorer in L2 than L1 for both phonological and semantic tasks. Especially, they showed poorer performance during the L2 phonological processing (lower accuracy and slower RT). Brain activation analysis demonstrated that L2 processing evoked more widespread and stronger activation in brain regions involved in auditory word processing across the tasks. In comparison between the PT and ST, K–E bilinguals revealed additional activation in bilateral SMG and the right frontal regions during L2 phonological processing. Furthermore, L2 evoked up-regulation in auditory processing regions was associated with individuals’ L2 proficiency. This is the first study demonstrates that K–E bilinguals showed increased FC in brain networks in L2 processing and the different sets of intra- and internetwork connectivity were involved in two different processing in L2 auditory word comprehension. FC analysis showed the increased intra-network connectivity in the LN, DAN, and DMN during L2 processing. Further analyses revealed that the intra-network connectivity within the DAN during phonological processing predicted better L2 proficiency. In contrast, for semantic processing, we found the enhanced connectivity between the LN and DMN. Our findings shed new light on the understanding of the second language comprehension by showing that L2 processing was supported by the enhanced FC in a language-specific network as well as domain general networks and the different features of tasks were involved indifferent sets of the intra-and internetwork connectivity. Furthermore, individual difference analyses suggest that the increased regional activity in key language regions and FC in brain networks contribute to individuals’ L2 proficiency.

Our results of brain activation patterns show that both L1 and L2 activated similar brain regions involved in auditory language comprehension including the IFG, STG, precentral gyrus, SMA, SMG, and IPL (Price, 2000) and L2 processing required greater activation in these regions in K–E bilinguals. Previous studies have reported the increased regional activity in language-specific regions in L2 processing (Kim et al., 1997; Perani et al., 2003; Wartenburger et al., 2003; Jones et al., 2012). Jones et al. (2012) examined the neural activity in bilinguals compared to monolinguals during picture naming and word reading. They found the increased activation in key language regions (the left frontal and temporal regions) when bilinguals performed both L2 tasks. Meschyan and Hernandez (2006) investigated neural representation in Spanish–English bilinguals with low L2 proficiency during single word reading. They compared the regional activity in L1 and L2 processing and found significant activations in brain regions involved in articulatory system including the SMA, STG, and right IPL during less fluent language processing. These studies suggest that the less practiced, less proficient, language requires greater regional activation. Consistent with these findings, our results showed that bilinguals with medium proficiency required greater regional activity in common regions involved in L1 processing. Also, we found that L2 phonological processing evoked additional activation in the bilateral SMG and the right frontal regions and significantly increased activation in the left IFG, STG, SMA and bilateral SMG and IPL in the ROI analysis. These regions are involved in controlling semantic interference and articulatory sequence (IFG) (Abutalebi, 2008; Jones et al., 2012), speech-motor preparation (SMA) (Klein et al., 1995; Riecker et al., 2005), auditory processing of speech (STG) (Britton et al., 2009; Jones et al., 2012), auditory processing of phonological features (pre/postcentral gyrus) (Booth et al., 2004), and auditory-articulatory interface system (SMG/IPL) (Callan et al., 2004; Chen and Desmond, 2005). K–E bilinguals performed the L2 PT poorer than any other conditions by showing lower accuracy and slower RT compared to L1 PT. Therefore, L2 phonological processing, which is less proficient, requires more involvement of the articulatory system in K–E bilinguals. Importantly, our results demonstrated that the regional activity modulated by L2 processing was associated with individuals’ L2 proficiency. K–E bilinguals showing greater activation in key regions of auditory word comprehension achieved higher scores in the official English test. This is a novel finding in our study that the increased regional activation in language regions can be beneficial for L2 performance in K–E bilinguals with medium proficiency.

One of the most important contributions of the current study is the findings of FC modulation in bilinguals’ brain networks during L2 processing. We found that K–E bilinguals showed greater intra-network connectivity in the LN and other domain general networks including the DAN and DMN for L2 processing. Previous studies reported that bilinguals had stronger FC between task-related regions (Cao et al., 2014; Li et al., 2015). Cao et al. (2014) demonstrated that Chinese–English bilinguals used the same network for L1 and L2 rhyming judgment and they had the increased connectivity between visual-orthographic regions and the left postcentral gyrus during English rhyming task. They argued that the somatosensory information of the foreign phonemes from the left postcentral gyrus was more encoded in the reading network compared to native language processing. Similarly, we found the enhanced FC in the LN for both L2 phonological and semantic processing. The LN consisting of the left IFG and the temporoparietal cortex was significantly activated for both phonological and semantic processing in both L1 and L2. And ROI analysis results revealed the increased regional activation in these regions in L2 compared to L1 across the tasks. Thus, our results support that the language system is shared for both L1 and L2 and is up-regulated at regional activity as well as FC for L2 auditory word comprehension in K–E bilinguals.

We found that K–E bilinguals showed the increased FC in the DAN during L2 phonological processing. The DAN is involved in mediating the top–down guided voluntary allocation of attention to sensory inputs (Vossel et al., 2014). It has been demonstrated that the connectivity between the key regions of the DAN (e.g., FEF and IPS) was greater when top-down modulation influences bottom–up sensory stimulation in attention (Bressler et al., 2008; Vossel et al., 2014). Our results are consistent with these studies in that L2 phonological processing induced the enhanced FC between FEF and IPS, because a phonological feature in Korean words (a long vowel) are more frequently encountered than that of English words (lexical stress) for our K–E bilinguals. Therefore, K–E bilinguals consciously might allocate their attention to less proficient sensory-based language features to perform our L2 PT. Moreover, bilinguals with stronger DAN FC during L2 phonological processing showed better L2 proficiency. To understand a spoken word, it is essential to process phonological features of a word. For example, young individuals with higher performance in phonological tasks showed better language comprehension (Engen and Høien, 2002; Cárnio et al., 2017). Likewise, L2 phonological skills can be important for understating L2 words. Thus, L2 phonological skills in K–E bilinguals can be beneficial in L2 word comprehension, which might contribute to their L2 proficiency. Our study is the first that provide evidence for the task-specific involvement of the DAN in L2 auditory word comprehension.

Different from L2 phonological processing, we found that L2 semantic processing was involved in the DMN, particularly in relation to the LN. The DMN is deactivated during specific goal-directed tasks and is involved in internally focused tasks including autobiographical memory retrieval, self-related thinking, and consciousness (Buckner et al., 2008). Also, studies in bilinguals with lifelong bilingual experience have reported the increased FC in the DMN compared to monolinguals (Luk et al., 2011; Grady et al., 2015). These studies suggest that stronger FC in the DMN is in line with the better maintained white-matter connections found in these bilinguals. Our results showed greater FC in the DMN in young bilinguals, which cannot be explained by changes underlying white matter pathways driven from lifelong experiences (e.g., older bilinguals in previous studies had about 30 years or more bilingual experience with high L2 proficiency). Furthermore, we found that L2 semantic processing induced stronger the internetwork connectivity between the DMN and LN. Recent studies reported that the DMN are involved in semantic processing (Wirth et al., 2011; Seghier and Price, 2012). A study investigating the DMN demonstrated that all key areas in the DMN showed reduced deactivation during language tasks than non-language tasks and part of the DMN in the mPFC and PCC were activated during semantic processing (Seghier and Price, 2012). Especially, they showed that the level of difficulty in semantic processing modulated the deactivation/activation of the DMN. They suggest that, when the brain is engaged in effortful semantic tasks, the DMN seems to interact with the semantic system. As K–E bilinguals with medium proficiency found that L2 semantic processing is more demanding than L1 (slower RT in L2 ST compared to L1 ST), it appears that the connectivity between the DMN and the LN plays a key role in semantic processing of English words. Patients with Alzheimer’s dementia or mild cognitive impairments also have demonstrated the involvement of the DMN in relation to their bilingual experience (Calvo et al., 2015; Perani et al., 2017). Bilingual patients showed the enhanced connectivity within and between the DMN and other task-active/related networks and this enhanced FC contributed to cognitive reserve in memory. Taken together, our results revealed that bilinguals with medium proficiency enhanced the connectivity within and between the DMN and the LN for L2 semantic processing. This is a novel finding that showed the task-specific integration between the DMN and task-related network in L2 auditory word comprehension.

It is noted that we did not find any significant differences in the ECN and SN between L1 and L2 processing. One possible explanation is that our bilinguals might have the enhanced ECN which is involved in both L1 and L2 processing. Previous studies have reported the increased involvement of these networks in bilinguals compared to monolinguals (Bialystok et al., 2012; Grady et al., 2015). Therefore, within-subjects comparisons cannot discriminate the changes within these networks. However, whole brain analyses demonstrated the significant activation in prefrontal cortex and parietal cortex as key regions of the ECN as well as insular and subcortical region as parts of SN during L2 processing in K–E bilinguals.

## Conclusion

Our study provide the first evidence that, even if the same brain networks are involved in L1 and L2 auditory word comprehension, FC in these networks are differently modulated by L2 tasks in K–E bilinguals. During L2 phonological processing, the DAN was interconnected strongly and this enhanced connectivity was associated with bilinguals’ L2 proficiency. L2 semantic processing strengthened the connectivity between the DMN and the LN, suggesting the involvement of the DMN in more demanding semantic processing in bilinguals. Our findings suggest that L2 processing in K–E bilinguals induces dynamic changes in the brain at a regional and network-level and FC analysis can disentangle the different networks involvement in L2 auditory word processing according to two key features: phonology and semantics. In K–E bilinguals, L2 processing related neural alterations may contribute to their L2 proficiency.

## Author Contributions

HC, SK, and KN: substantial contributions to the conception or design of the work. JJ and HC: the acquisition, analysis of data for the work. JJ, SK, and KN: the interpretation of data for the work. JJ and SK: drafting the work or revising it critically for important intellectual content. JJ and SK: final approval of the version to be published.

## Conflict of Interest Statement

The authors declare that the research was conducted in the absence of any commercial or financial relationships that could be construed as a potential conflict of interest. The reviewer SF and handling Editor declared their shared affiliation.

## Footnotes

1 www.ansir.wfubmc.edu

2 http://web.mit.edu/swg/software.htm
