# Supplementary material for: Mapping the Neural Dynamics of Korean–English Bilinguals With Medium Proficiency During Auditory Word Processing
Source: Front Psychol. 2018 Jun 18;9:983. doi: 10.3389/fpsyg.2018.00983 (PMC6015918; doi:10.3389/fpsyg.2018.00983)
Supplement: Supplementary file 1 [file Table_1.DOCX]

**Supporting information**

**Table S1** Stimuli

| Language | Task | Item | Meaning | Task | Item | Meaning | Language | Task | Item | Task | Item |
| --- | --- | --- | --- | --- | --- | --- | --- | --- | --- | --- | --- |
| L1 | Phonological processing | 가격 | price | Semantic Processing | 가:짜 | fake | L2 | Phonological processing | circle | Semantic Processing | guilty |
|  |  | 무릎 | knee |  | 거:절 | refusal |  |  | release |  | passion |
|  |  | 비누 | soup |  | 도박 | gambling |  |  | cartoon |  | crisis |
|  |  | 소금 | salt |  | 대:립 | conflict |  |  | double |  | dessert |
|  |  | 사진 | picture |  | 뇌물 | bribe |  |  | command |  | correct |
|  |  | 가방 | bag |  | 도둑 | thief |  |  | listen |  | damage |
|  |  | 시간 | time |  | 비:극 | tragedy |  |  | traffic |  | justice |
|  |  | 자리 | room |  | 사:망 | death |  |  | routine |  | conflict |
|  |  | 머리 | head |  | 시:련 | ordeal |  |  | distinct |  | success |
|  |  | 소리 | sound |  | 무덤 | grave |  |  | blanket |  | cigar |
|  |  | 부엌 | kitchen |  | 자살 | suicide |  |  | detect |  | neglect |
|  |  | 가족 | family |  | 거:지 | beggar |  |  | fiction |  | respect |
|  |  | 기름 | oil |  | 노:화 | aging |  |  | respond |  | precious |
|  |  | 바지 | pants |  | 고통 | pain |  |  | purchase |  | victim |
|  |  | 저녁 | evening |  | 비:만 | obesity |  |  | distance |  | pleasure |
|  |  | 비:서 | secretary |  | 미:모 | beauty |  |  | finance |  | wisdom |
|  |  | 사:십 | forty |  | 다행 | lucky |  |  | conscious |  | murder |
|  |  | 자:세 | posture |  | 사랑 | love |  |  | combine |  | tragic |
|  |  | 자:랑 | boast |  | 화해 | reconciliation |  |  | measure |  | magic |
|  |  | 수:건 | towel |  | 가:망 | prospect |  |  | portion |  | regret |
|  |  | 비:밀 | secret |  | 사탕 | candy |  |  | discuss |  | suffer |
|  |  | 가:게 | store |  | 다정 | tenderness |  |  | concept |  | belief |
|  |  | 서:술 | description |  | 저:택 | mansion |  |  | collect |  | severe |
|  |  | 세:상 | world |  | 미:덕 | virtue |  |  | machine |  | silver |
|  |  | 주:소 | address |  | 저:축 | saving |  |  | select |  | burden |
|  |  | 서:론 | introduction |  | 고향 | hometown |  |  | bottom |  | journey |
|  |  | 수:박 | watermelon |  | 마:법 | magic |  |  | budget |  | profit |
|  |  | 도:착 | arrival |  | 새벽 | dawn |  |  | report |  | divorce |
|  |  | 소:변 | urine |  | 희망 | hope |  |  | result |  | plenty |
|  |  | 비:교 | comparison |  | 노력 | effort |  |  | patient |  | disturb |
